# Gender Differences in How the American Public Reacts to a Person With Mild-Stage Dementia

**DOI:** 10.1093/geroni/igaa057.2696

**Published:** 2020-12-16

**Authors:** Shana Stites, Emily Largent, Jeanine Gill, Rebecca Johnson, Kristin Harkins, Jason Karlawish

**Affiliations:** 1 University of Pennsylvania, Philadelphia, Pennsylvania, United States; 2 Princeton University, Princeton, New Jersey, United States

## Abstract

Many studies show that caregivers for those with Alzheimer’s Disease (AD) are disproportionately female, but few studies have investigated how public attitudes influence this gender disparity. We analyzed secondary data from an experimental study of public reactions to AD dementia. Analysis included 944 respondents who read a vignette about a man with mild stage dementia and completed a modified Family Stigma in Alzheimer’s Disease Scale (FS-ADS), which assesses 7 domains of stigma. Multivariable ordered logistic regression compared men and women on FS-ADS ratings. Women were less likely than men to endorse stronger negative aesthetic attributions (OR=0.75) and negative feelings (OR=0.76) and more likely to endorse stronger feelings of pity (OR=1.33; all p<0.05). No other differences were observed in FS-ADS domains (all p>0.05). The findings offer insights into relationships between gender and AD stigma, which may influence who is willing to become a caregiver for persons with AD and related dementias.

